# Cardiovascular effects of small peptides of the renin angiotensin system

**DOI:** 10.14814/phy2.13505

**Published:** 2017-11-28

**Authors:** Patrícia L. Moraes, Lucas M. Kangussu, Luiz Gonzaga da Silva, Carlos H. Castro, Robson A.S. Santos, Anderson J. Ferreira

**Affiliations:** ^1^ Department of Morphology Biological Sciences Institute Federal University of Minas Gerais Belo Horizonte Brazil; ^2^ Department Physiology and Biophysics Biological Sciences Institute Federal University of Minas Gerais Belo Horizonte Brazil; ^3^ Life Sciences Institute Federal University of Juiz de Fora Governador Valadares Brazil; ^4^ Department of Physiological Sciences Federal University of Goiás Goiânia Brazil

**Keywords:** Angiotensin‐(1‐2), Small peptides, Vasodilation

## Abstract

The renin‐angiotensin system (RAS) is a unique hormonal cascade which is composed by multiple enzymes and effector peptides. Recently, new peptides presenting biological activity have been discovered, increasing the complexity of the RAS. Here, we evaluated the effects of small peptides of the RAS in coronary bed of rats. Firstly, we examined the direct effect of small angiotensinergic peptides [Angiotensin (Ang) ‐(1–5), Ang‐(1–4) Ang‐(1–3), and Ang‐(1–2)] in coronary vessels. Noteworthy, it was observed that Ang‐(1–4), Ang‐(1–3), and Ang‐(1–2) caused a significant reduction in pressure perfusion. Because Ang‐(1–2) was the smallest peptide tested and presented the major effect, we decided to investigate its mechanisms of action. The effect of Ang‐(1–2) was partially dependent on the Mas receptor, nitric oxide release and angiotensin‐converting enzyme. Importantly, Ang‐(1–2) reduced the blood pressure of Wistar rats and SHR. Interestingly, SHR presented a more pronounced decrease in blood pressure levels than Wistar rats. Altogether, these data showed that angiotensinergic small peptides hold biological activities in coronary bed of rats.

## Introduction

The renin‐angiotensin system (RAS) is a complex hormonal cascade which is composed by multiple enzymes and effector peptides (Lavoie and Sigmund 2003; Paul et al. 2006; Fyhrquist and Saijonmaa 2008). The classical axis of the RAS, formed by angiotensin converting enzyme (ACE), Angiotensin (Ang) II and AT_1_ receptor, activates several cell functions and when hyperactivated causes deleterious effects, including hypertension, fibrosis, cardiac and vascular hypertrophy, endothelial dysfunction and vasoconstriction. On the other hand, the counter‐regulatory axis composed by ACE2, Ang‐(1–7), and Mas receptor exerts opposite effects, such as antihypertrophic and antifibrotic effects, improvement of endothelial function, nitric oxide release and vasodilation in several vascular beds (Lazartigues et al. 2007; Santos et al. 2008, 2013; Bader 2010; Shahid 2011).

Recently, new peptides holding biological activity have been discovered and this phenomena has added more complexity to the RAS (Ohishi et al. 2013; Etelvino et al. 2014). For instance, removing one amino acid from the amino‐terminal end of the Ang II by aminopeptidase A, Ang III (Arg^2^‐Val^3^‐Tyr^4^‐Ile^5^‐His^6^‐Pro^7^‐Phe^8^) is formed, which in turn can be cleaved by aminopeptidase N forming Ang IV (Val^3^‐Tyr^4^‐Ile^5^‐His^6^‐Pro^7^‐Phe^8^). More recently, Ang A (Ala^1^‐Arg^2^‐Val^3^‐Tyr^4^‐Ile^5^‐His^6^‐Pro^7^‐Phe^8^) which is formed by decarboxylation of the aspartate amino acid of the Ang II sequence by aspartate decarboxylase, and Alamandine (Ala^1^‐Arg^2^‐Val^3^‐Tyr^4^‐Ile^5^‐His^6^‐Pro^7^) produced by ACE2 from Ang A were also described (Jankowski et al. 2007; Lautner et al. 2013). In addition, smaller fragments, such as Ang‐(5–8) (Ile^5^‐His^6^‐Pro^7^‐Phe^8^), Ang‐(4–8) (Tyr^4^‐Ile^5^‐His^6^‐Pro^7^‐Phe^8^), Ang‐(2–7) (Arg^2^‐Val^3^‐Tyr^4^‐Ile^5^‐His^6^‐Pro^7^), Ang‐(3–7) (Val^3^‐Tyr^4^‐Ile^5^‐His^6^‐Pro^7^), Ang‐(1–5) (Asp^1^‐Arg^2^‐Val^3^‐Tyr^4^‐Ile^5^), Ang‐(1–4) (Asp^1^‐Arg^2^‐Val^3^‐Tyr^4^), Ang‐(3–4) (Val^3^‐Tyr^4^), as well as the enzymes involved in their formation, are for long time recognized. However, their biological effects have not been explored so far (Matsufuji et al. 1995; Ardaillou, 1997; Axelband et al. 2009).

Thus, the importance and complexity of the RAS have been increasingly documented. Evaluation of function and mechanisms of action involved in the biological activity of small and poorly studied peptides is an important step toward a better understanding of this system. The description of new biologically active peptides of the RAS can change the paradigm that peptides derived from the metabolism of larger peptides like Ang II and Ang‐(1–7) are inactive and irrelevant fragments. Instead, they may be of great physiological meaning and important pharmacological targets for future interventions in this system. Therefore, the aim of this study was to evaluate the effects of small peptides of the RAS in the cardiovascular system.

## Methods

### Animals and ethical approval

Experiments were performed in male Wistar rats (250–300 g) from the Animal Facility (CEBIO) of the Federal University of Minas Gerais (UFMG), Brazil, and in spontaneously hypertensive rats (SHR) (16 weeks of age) supplied by the animal facilities of the Laboratory of Hypertension (UFMG, Brazil). The animals were housed 4–5 per cage and allowed free access to food and water in controlled environment conditions (temperature 22–23°C and 12 h:12 h light‐dark cycles). All experimental procedures were approved by the Committee for Ethics in Animal Experimentation of UFMG (Protocol 051/2010).

### Isolated heart preparation

The rats were killed by decapitation 10–15 min after intraperitoneal injection of heparin (400 IU). The thorax was opened and the heart was carefully dissected and perfused through a 1.0 ± 0.3 cm aortic stump with Krebs‐Ringer solution (KRS) containing (in mmol/L): 118.4 NaCl, 4.7 KCl, 1.2 KH_2_PO_4_, 1.2 MgSO_4_. 7H_2_O, 2.5 CaCl_2_. 2H_2_O, 11.7 glucose, and 26.5 NaHCO_3_. The perfusion flow was maintained constant (8–9 mL/min) at 37 ± 1°C with constant oxygenation (5% CO_2_ and 95% O_2_). A pressure transducer (Biopac Systems, Santa Barbara, CA) was attached to the aortic stump to record the perfusion pressure and a force transducer (Biopac Systems, Santa Barbara, CA) was attached through a heart clip to the apex of the ventricles to record the cardiac contractility on a computer through a data‐acquisition system (Biopac Systems, Santa Barbara, CA). A basal tension of 0.5–1.0 g was applied to the hearts. The heart rate (HR) and ±dT/dt were calculated from the contraction records. After a stabilization period of 30 min, the perfusion solution was changed to KRS containing the peptides and/or antagonists/inhibitors. Afterward, an additional period of ~30 min was allowed to record the effects of the drugs.

Initially, we tested the effects of Ang‐(1–5) (42 pmol/L, *n *=* *5), Ang‐(1–4) (42 pmol/L, *n *=* *5), Ang‐(1–3) (42 pmol/L, *n *=* *6), Ang‐(1–2) (42 pmol/L, *n *=* *6), and L‐arginine (42 pmol/L, *n *=* *7). Since we observed that Ang‐(1–2) produced the larger reduction in the perfusion pressure among the peptides tested (see Results below), we focused on the investigation of the mechanisms related to the vasodilatory effect of Ang‐(1–2). Then, we tested the perfusion of Ang‐(1–2) in combination with A779, a Mas receptor antagonist (23 nmol/L, *n *=* *7), captopril, an ACE inhibitor (2.5 *μ*mol/L, *n *=* *4) and infusion of Ang‐(1–2) in hearts of rats previously treated with NG‐nitro‐L‐arginine methyl ester (L‐NAME), a nonselective nitric oxide synthase (NOS) inhibitor (i.p. 30 min before the sacrifice; 30 mg/kg; *n *=* *5). Control hearts (*n *=* *6) were perfused all the time with KRS alone. Inhibitors and antagonists concentrations were defined based on previous results from our laboratory and published studies (Moltzer et al. 2010; Souza et al. 2013; Wołkow et al. 2016; Yu et al. 2016; Moraes et al. 2017).

### Blood pressure and heart rate analysis

To further demonstrate the biological activity of Ang‐(1–2), we also tested the effect of the Ang‐(1–2) on mean arterial pressure (MAP) and heart rate (HR) in conscious normotensive rats and in SHR. Wistar rats (*n *=* *5) and SHR (*n *=* *5) were submitted to a surgery for implantation of a femoral catheter. Under anesthesia with a 10% ketamine and 2% xylazine mixture (60:6 mg/kg, i.p.), a catheter (PE‐10 connected to a PE‐50) was inserted into the femoral artery (until abdominal aorta) and femoral vein for blood pressure measurement and peptide injections, respectively. The catheters were tunneled subcutaneously into the back of the neck to allow access when the animal was awake. After 24 h of recovery, basal MAP and HR were evaluated in nonanesthetized animals. The arterial catheter was connected to a strain‐gauge transducer coupled to a computer‐based data acquisition system (MP100A, Biopac Systems Inc., CA) in order to record pulsatile arterial pressure (PAP). MAP and HR were simultaneously calculated by the software Acqknowledge (Biopac Systems Inc., CA) and continuously displayed. After 15 min of stabilization, a control injection (0.1 mL of saline) was made through the femoral vein. Ang‐(1–2) (20 nmol) was administered in bolus of 0.1 mL after 30 min of the injection of saline and an additional 10‐min period was recorded. The basal values of MAP and HR were established 2 min before saline injection and all analyses were made in each interval of 2 min (ΔMAP, mmHg and ΔHR, bpm).

### Statistical analysis

All data are expressed as mean ± SEM and the level of significance was set at *p*< 0.05. Statistical analyses were performed using paired or unpaired Student's *t*‐test or One‐way ANOVA followed by the Newman‐Keuls *post hoc* test, as specified in each table or figure legend. Wilcoxon test or Mann‐Whitney test followed by the Dunns' *post hoc* test were used for non‐parametric data. The effect of each treatment was calculated by the difference between the values before and after the perfusion of the drugs. Graphic plotting and statistical analysis were performed using Graphpad Prism software (version 5.0, La Jolla, CA, USA).

## Results

**Figure 1:**
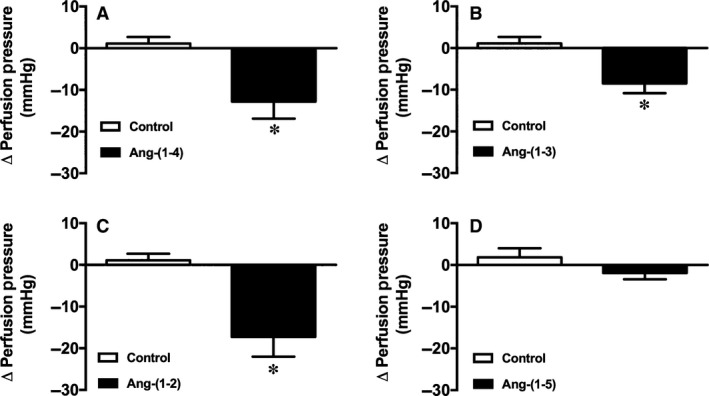
Effects of angiotensinergic peptides on coronary vessels of isolated rat hearts. (A) Ang‐(1–4), (B) Ang‐(1–3), and (C) Ang‐(1–2) perfusion caused a significant reduction in the perfusion pressure. (D) Perfusion of Ang‐(1–5) did not cause any significant alteration in perfusion pressure. Data are shown as mean ± S.E.M. * *P* < 0.05 versus control. Unpaired Student's *t*‐test or Mann‐Whitney's test.

The first experiment was designed to test the effects of different small peptides of the RAS in coronary bed of rats. It was observed that, in isolated rat hearts submitted to perfusion with constant flow, Ang‐(1–4) [Control: 1.12 ± 1.56 mmHg and Ang‐(1–4): −12.81 ± 4.07 mmHg, *P* < 0.05; Fig. 1A], Ang‐(1–3) [Control: 1.12 ± 1.56 mmHg and Ang‐(1–3): −8.47 ± 2.35 mmHg, *P* < 0.05; Fig. 1B] and Ang‐(1–2) [Control: 1.12 ± 1.56 mmHg and Ang‐(1–2): −17.27 ± 4.75 mmHg, *P* < 0.05; Fig. 1C] significantly decreased the perfusion pressure, indicating a vasodilatory action of these small peptides. Differently, Ang‐(1–5) perfusion did not promote any significant change in the perfusion pressure [Control: 1.12 ± 1.56 mmHg and Ang‐(1–5): −1.92 ± 1.49 mmHg; Fig. 1D].

We also analyzed the peptides effects on HR and ventricular contractility, that is, systolic and diastolic tensions, maximal rate of ventricular tension rise (+dT/dt), and maximal rate of ventricular tension decline (−dT/dt). Basal values were compared with values after infusion of the peptides. Table 1 shows that Ang‐(1–4) caused systolic tension decrease and diastolic tension increase in isolated hearts. Ang‐(1–3) and Ang‐(1–2) did not promote any significant changes in diastolic tension, but they decreased systolic tension. Furthermore, Ang‐(1–4), Ang‐(1–3), and Ang‐(1–2) reduced +dT/dt and −dT/dt. None of these peptides altered the HR and Ang‐(1–5) did not cause any alteration in these parameters.

**Table 1:** ‐ Effects of small angiotensinergic peptides on cardiac function

	Ang‐(1–5)		Ang‐(1–4)		Ang‐(1–3)		Ang‐(1–2)	
	Before	After	Before	After	Before	After	Before	After
Systolic tension (g)	6.91 ± 1.34	6.51 ± 1.22	10.47 ± 1.19	8.31 ± 1.06^a^	9.85 ± 0.74	8.17 ± 0.74^a^	10.40 ± 1.32	9.44 ± 1.41^a^
Diastolic tension (g)	0.94 ± 0.02	0.95 ± 0.03	0.86 ± 0.02	0.99 ± 0.03^a^	0.93 ± 0.01	0.98 ± 0.02	0.85 ± 0.03	1.07 ± 0,10
+ dT/dt (g/min)	97.34 ± 24.21	92.43 ± 21.64	154.10 ± 20.13	124.80 ± 17.66^a^	132.50 ± 9.95	115.20 ± 10.52^a^	153.90 ± 23.85	136.40 ± 24.52^a^
‐dT/dt (g/min)	100.10 ± 17.57	95.78 ± 16.22	135.20 ± 14.28	88.72 ± 13.81^a^	112.10 ± 10.43	83.06 ± 6.64^a^	140.50 ± 23.84	118.30 ± 25.63^a^
Heart rate (bpm)	228.10 ± 7.78	232.00 ± 11.21	207.60 ± 10.88	194.50 ± 10.32	191.20 ± 7.38	193.70 ± 9.78	207.40 ± 12.21	206.00 ± 11.89

Data are shown as mean ± SEM.

a *P* < 0.05 versus before perfusion of the respective peptide. Paired Student's *t*‐test or paired Wilcoxon's test.

**Figure 2:**
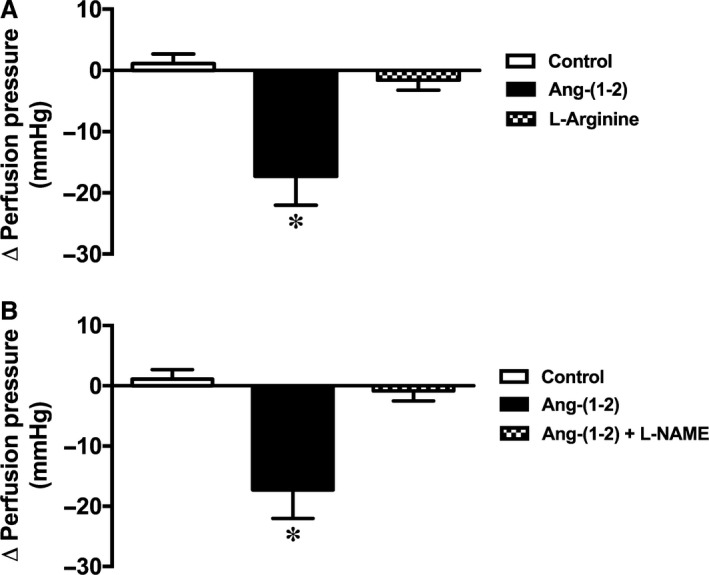
Effect of L‐arginine on coronary vessels of isolated rat hearts and the role of NO release in the Ang‐(1–2) vasodilator effect. (A) L‐Arginine did not cause any significant change in the perfusion pressure. (B) L‐NAME completely abolished the Ang‐(1–2) effect on the coronary perfusion pressure of isolated rat hearts. Data are shown as mean ± S.E.M. * *P* < 0.05 versus all other groups (One‐way ANOVA followed by Newman‐Keuls *post hoc* test).

**Figure 3:**
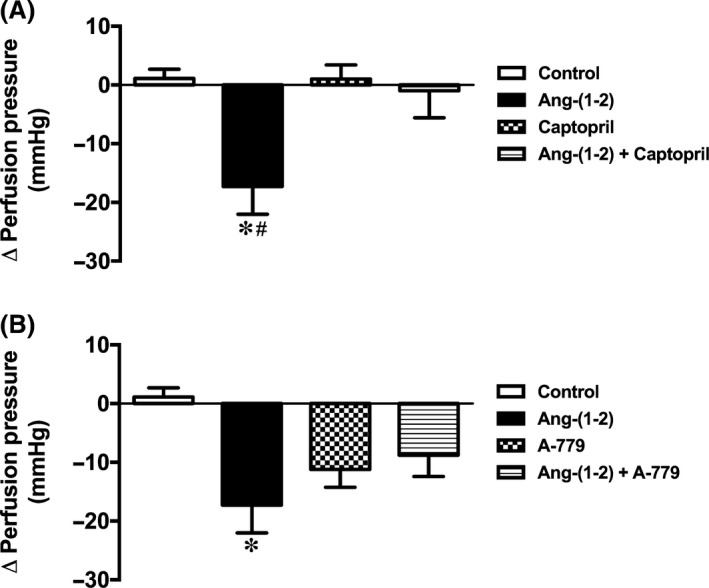
Effects of angiotensin‐converting enzyme and Mas receptor on the Ang‐(1–2) vasodilator effect. (A) captopril completely abolished and (B) A779 reduced the Ang‐(1–2) effect on the coronary perfusion pressure of isolated rat hearts. Data are shown as mean ± S.E.M. * *P* < 0.05 versus control and ^#^ *P* < 0.05 versus captopril or Ang‐(1‐2) + captopril. (One‐way ANOVA followed by Newman‐Keuls *post hoc* test).

Subsequently, we evaluated the mechanisms of action of Ang‐(1–2) in the coronary bed, since it was the smallest peptide with the amino‐terminal extremity conserved. Firstly, L‐arginine, a vasoactive amino acid that is one of the two amino acids of the Ang‐(1–2), was tested. As showed in Figure 2A, L‐arginine did not elicit any significant change in the perfusion pressure [Control: 1.12 ± 1.56 mmHg and L‐arginine: −1.58 ± 1.62 mmHg; Fig. 2A], showing that L‐arginine alone is unable to induce vasodilation in the coronary bed. Next, we assessed the participation of nitric oxide (NO) in the Ang‐(1–2) effects. The NOS inhibitor, L‐NAME, completely abolished the coronary vasodilation induced by this peptide [control: 1.12 ± 1.56 mmHg; Ang‐(1–2): −17.27 ± 4.75 mmHg; Ang‐(1–2) plus L‐NAME: −0.86 ± 1.66 mmHg, *P* < 0.05; Fig. 2B]. In addition, we evaluated the participation of ACE and Mas in the Ang‐(1–2) effects. Captopril also blunted the Ang‐(1–2) effect [control: 1.12 ± 1.56 mmHg; Ang‐(1–2): −17.27 ± 4.75 mmHg; Captopril: 1.03 ± 2.39 mmHg; Ang‐(1–2) plus captopril: −0.98 ± 4.59 mmHg, *P* < 0.05; Fig. 3A]. Similar effects were observed when A779 was used in addition to Ang‐(1–2) perfusion [control: 1.12 ± 1.56 mmHg; Ang‐(1–2): −17.27 ± 4.75 mmHg; A779: −11.21 ± 3.03 mmHg; Ang‐(1–2) plus A779: −8.78 ± 3.64 mmHg, *P* < 0.05; Fig. 3B].

**Figure 4:**
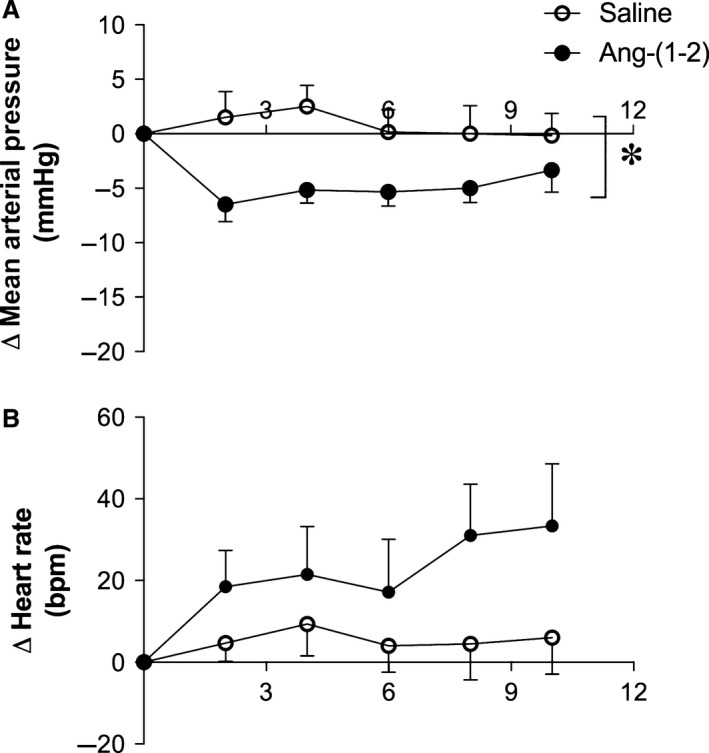
Effects of Ang‐(1–2) infusion on mean arterial pressure (MAP) and heart rate (HR) of conscious Wistar rats among time (minutes). Ang‐(1–2) infusion reduced the (A) MAP but did not alter the (B) HR. Data are shown as mean ± S.E.M. * *P* < 0.05 versus control. Unpaired Student's *t*‐test.

**Figure 5:**
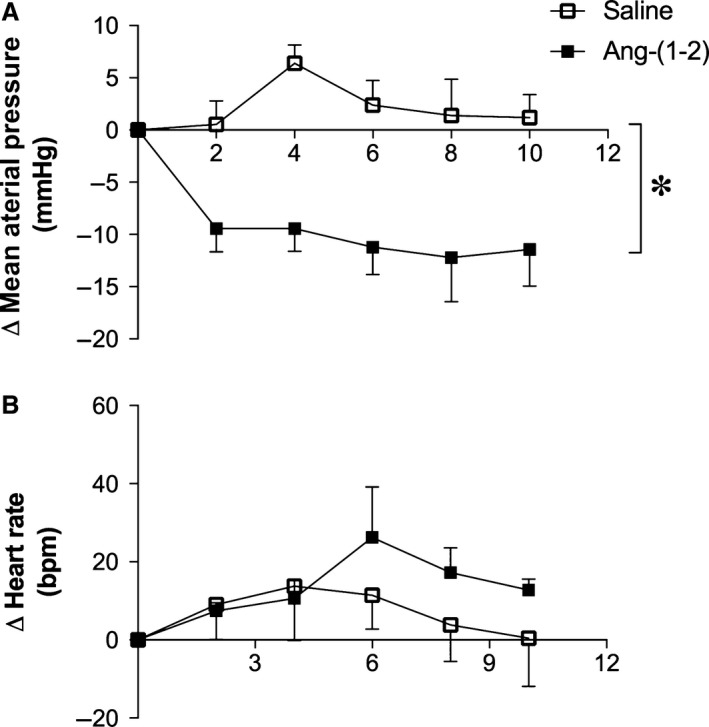
Effects of Ang‐(1–2) infusion on mean arterial pressure (MAP) and heart rate (HR) of awake SHR among time (minutes). Ang‐(1–2) infusion reduced the (A) MAP but did not alter the (B) HR. Data are shown as mean ± S.E.M. * *P* < 0.05 versus control. Unpaired Student's *t*‐test.

In vivo experiments showed that Ang‐(1–2) injection into femoral vein of conscious rats induced a significant decrease in MAP of Wistar rats and SHR when compared with saline injection (*P* < 0.05; Figs. 4 and 5, respectively). SHR presented a more pronounced decrease in blood pressure levels than Wistar rats (mean changes −8.9 ± 1.8 *vs*. −4.2 ± 0.9 mmHg, respectively). Also, the decrease in MAP in SHR after Ang‐(1–2) injection lasted for at least 30 min, while in Wistar rats MAP returned to baseline levels after ~10 min (data not shown). No significant changes in HR were viewed after Ang‐(1–2) injection (Figs. 4 and 5).

## Discussion

The major findings of this study are that small peptides of the RAS [Ang‐(1–4), Ang‐(1–3) and Ang‐(1–2)] induced a reduction in the perfusion pressure of isolated hearts, indicating vasodilation in coronary bed of rats. Because Ang‐(1–2) was the smallest peptide tested and presented the major effect, we decided to investigate its mechanisms of action. We found that this effect was mediated by NO release, ACE and partially dependent on Mas activation. Moreover, Ang‐(1–2) reduced the blood pressure of conscious normotensive and hypertensive rats.

We found that Ang‐(1–4), Ang‐(1–3), and Ang‐(1–2) caused significant reduction in the perfusion pressure and altered the cardiac contractility of isolated rat hearts, indicating that these peptides hold biological actions in hearts. At the concentration tested, Ang‐(1–5) did not induce any significant effects in coronary arteries or on cardiac contractility. Besides that it has been reported that Ang‐(1–5) is an active peptide of RAS, since it stimulates ANP secretion via Mas and PI3K‐Akt‐NOS pathway (Yu et al. 2016). This divergent result likely is related to differences in protocols and concentrations of the peptide used in these studies.

Because Ang‐(1–2) was the smallest peptide tested with the amino‐terminal extremity conserved, we focused our efforts on this peptide. Initially, we evaluated the coronary effect of L‐arginine, a vasoactive amino acid that composes the amino acids sequence of Ang‐(1–2) and a substrate for NO formation (Bian and Murad 2003). In our preparation, this amino acid did not induce any significant effects on perfusion pressure, thereby discarding the possibility that the vasodilator action of Ang‐(1–2) was due to its degradation with L‐arginine release.

Afterward, we evaluated the mechanisms of action of Ang‐(1–2) in the coronary bed. It was verified that Ang‐(1–2) exerts its effects by binding to Mas since vascular actions of this dipeptide were attenuated by A779. This result is an indicative that the first two amino acids of the Ang‐(1–7) sequence are important to its interaction with Mas. In addition, the finding showing that L‐NAME abolished the Ang‐(1–2) effects indicates that this dipeptide plays its actions through a mechanism mediated by NO release. Surprisingly, ACE inhibition was also able to block the Ang‐(1–2) effects, indicating that more efforts must be done to elucidate the exact mechanism of actions of Ang‐(1–2). For instance, the role of AT_2_ and B_2_ receptors should be investigated in this context.

Recently, Brar et al. (2017) showed that Ang‐(1–2) is responsible for increasing the insulin secretion in vitro, a result previously attributed to Ang‐(1–7). They observed that both neprilysin and ACE2 are required for Ang‐(1–7) to enhance insulin secretion in vitro. Neprilysin cleaves Ang‐(1–7) to generate the insulinotropic Ang‐(1–2) dipeptide which acts, at least in part, through GPCR6A. This was probably the first report showing that intact Ang‐(1–7) is not the primary mediator of beneficial effects attributed to the ACE2/Ang‐(1–7)/Mas axis, but a peptide derived from its cleavage. GPCR6A should also be investigated in the coronary effects of Ang‐(1–2), since it can bind small peptides and L‐*α*‐amino acids, and stimulate the increase in intracellular calcium concentrations (Kuang et al. 2005; Pi et al. 2005; Wellendorph et al. 2005).

Importantly, Ang‐(1–2) reduced the MAP of conscious Wistar rats and SHR. The vasodilatory effect in SHR was more pronounced and lasted for at least 30 min, while Wistar rats recovered their baseline levels after 10 min. The compromised blood pressure regulatory mechanisms can be an explanation for these results in SHR (Head 1994; Heringer‐Walther et al. 2001). This augmented effect of Ang‐(1–2) in SHR is a positive element in the control of blood pressure of hypertensive subjects. More studies are required to clarify how the small angiotensin peptides are formed and if Ang‐(1–4) and Ang‐(1–3) can induce vasodilation in normotensive and hypertensive animals.

In summary, our present data showed that small fragments derived from the RAS hold biological activities in coronary bed. Among the peptides evaluated, the effects of higher intensity was observed using Ang‐(1–2), which induced its actions through ACE, NO release, and activation of Mas.

## Conflict of Interest

The authors declare that they have no competing interests.
